# RetinaDetachNet: Automated Deep Learning Quantification of Photoreceptor Cell Death for Neuroprotection Studies in Experimental Retinal Detachment

**DOI:** 10.1167/tvst.15.4.23

**Published:** 2026-04-22

**Authors:** Konstantinos G. Baroutis, Hani El Helwe, Kaho Yamamoto, Maria Anna Bantounou, Irini Chatziralli, Ilias Georgalas, Panagiotis Theodossiadis, Yusuke Murakami, Joan W. Miller, Demetrios G. Vavvas

**Affiliations:** 1 Department of Ophthalmology, Retina Service, Ines and Frederick Yeatts Lab in Retina Research, Massachusetts Eye and Ear, Harvard Medical School, Boston, MA, USA; 2 Department of Ophthalmology, Kyushu University Hospital, Fukuoka, Japan; 3 Department of Ophthalmology, National and Kapodistrian University of Athens, Athens, Greece

**Keywords:** retinal detachment, TUNEL assay, cell death quantification, deep learning, StarDist, automated image analysis, photoreceptor cell death, neuroprotection

## Abstract

**Purpose:**

To develop and validate RetinaDetachNet, to our knowledge, the first validated deep learning pipeline for automated quantification of TUNEL-positive cells in experimental retinal detachment models.

**Methods:**

RetinaDetachNet combines a custom-trained U-Net for outer nuclear layer (ONL) segmentation with a hybrid approach for TUNEL-positive cell detection. StarDist provides initial nucleus segmentation; candidates are retained only if they satisfy area criteria and overlap sufficiently with Otsu-thresholded binary masks. Validation involved three independent datasets with temporal and institutional separation: primary (*n* = 50 images), historical (∼10 years prior; *n* = 50), and external (independent laboratory; *n* = 40). Agreement with manual counts (experienced and inexperienced observers) was assessed via Spearman correlation (5000 bootstrap iterations) and Bland-Altman analysis.

**Results:**

The U-Net achieved a Dice coefficient of 0.93 for ONL segmentation. RetinaDetachNet showed strong correlation with manual counting: Dataset 1, ρ = 0.87 (experienced) and ρ = 0.80 (inexperienced); Dataset 2, ρ = 0.98 (surpassing inter-observer ρ = 0.94); Dataset 3, ρ = 0.86 following calibration to local imaging parameters. The hybrid method outperformed StarDist-only (ρ = 0.70–0.84) and Otsu-only (ρ = 0.33–0.90) approaches across datasets. Bland-Altman analysis indicated minimal systematic bias.

**Conclusions:**

To our knowledge, RetinaDetachNet is the first validated deep learning pipeline for TUNEL-positive cell quantification in retinal detachment, delivering superior accuracy and reproducibility via its hybrid dual-validation architecture.

**Translational Relevance:**

This open-source tool, freely available on GitHub, enables standardized, observer-independent quantification of photoreceptor cell death in preclinical neuroprotection studies, reducing analysis time from hours to minutes and accelerating evaluation of candidate therapeutics.

## Introduction

Retinal detachment (RD), a sight-threatening condition, involves the separation of photoreceptors (PR) from the underlying retinal pigment epithelium (RPE). Retinal detachment disrupts RPE-photoreceptor interactions, impairing pigment recycling, phagocytosis, and nutrient transport, triggering apoptosis, necroptosis, and autophagy in photoreceptors.^1^^–^^6^ The seminal work by Cook et al.^7^ first demonstrated apoptosis as the primary form of photoreceptor degeneration after RD using the TUNEL assay, establishing the foundational methodology for subsequent RD research. Caspase-independent pathways have since been characterized as additional mechanisms of photoreceptor death.^8^^,^^9^

The terminal deoxynucleotidyl transferase dUTP nick end labeling (TUNEL) assay remains the gold standard for quantifying cell death in the experimental RD model.^10^ The assay detects DNA fragmentation, a hallmark of apoptosis, though standardized nomenclature recognizes that TUNEL may also label cells undergoing other forms of regulated cell death.^11^ Although standardized TUNEL staining protocols have been established,^12^ quantifying TUNEL-positive cells (TUNEL+) traditionally relies on manual counting by trained observers, which is time-consuming, labor-intensive, and prone to inter- and intra-observer variability.^13^^–^^15^ To address these limitations, conventional image processing pipelines using thresholding and watershed segmentation have been developed.^10^^,^^16^ However, these traditional approaches face significant challenges when applied to complex, noisy, or variable medical images, often resulting in low accuracy and high noise sensitivity.^17^

The advent of deep learning has revolutionized biomedical image analysis, enabling improved accuracy in cell segmentation and quantification tasks.^17^^–^^19^ U-Net, introduced by Ronneberger et al.^20^ in 2015, established the foundation for modern medical image segmentation with its encoder-decoder architecture featuring skip connections that preserve both contextual and spatial information. The use of pretrained encoders (e.g., VGG, ResNet) with U-Net architectures has further improved segmentation performance by leveraging transfer learning from large-scale image datasets.^21^ Self-configuring approaches such as nnU-Net have since established benchmarks for automated medical image segmentation without manual parameter tuning.^22^ Content-aware image restoration techniques, including the CSBDeep framework, have complemented these advances by enabling robust image normalization for fluorescence microscopy applications.^23^

StarDist, developed by Schmidt et al.,^24^ represents a significant advancement in cell segmentation by using star-convex polygons to represent cell shapes, a representation well suited for the typically roundish morphology of cell nuclei in fluorescence microscopy. Built on a U-Net backbone, StarDist has demonstrated superior performance compared to traditional segmentation approaches across diverse applications, including circulating tumor cell detection (99.95% sensitivity with 10%–20% additional cell detection),^25^ three-dimensional (3D) nuclei segmentation in tissue context,^26^ and general fluorescence microscopy applications. An alternative approach, Cellpose, uses gradient flow tracking for generalist cellular segmentation and has shown strong performance across diverse cell types and imaging conditions.^27^^,^^28^ The 2018 Data Science Bowl established standardized benchmarks for evaluating nucleus segmentation algorithms, demonstrating the substantial performance gains achievable through deep learning approaches.^29^ The pretrained 2D_versatile_fluo model has been validated across multiple imaging modalities and cell types, demonstrating robust generalization capabilities.^24^^–^^26^

Despite advances in deep learning for cell death detection in related applications, methods specifically validated for TUNEL quantification in RD models remain absent from the literature, representing a significant research gap. Notably, although automated approaches have been developed for related tasks such as retinal ganglion cell quantification using deep learning,^30^ no validated pipeline exists for TUNEL+ cell quantification in experimental RD.

In this article, we develop RetinaDetachNet, an open-source Python application that leverages two deep learning architectures: a custom-trained U-Net for outer nuclear layer (ONL) segmentation and StarDist for cell detection, for automated quantification of TUNEL+ cells in experimental RD. RetinaDetachNet automates ONL recognition, TUNEL+ cell counting, and nuclei density calculation through a user-friendly graphical interface with tunable parameters. This tool enhances reproducibility, efficiency, and accessibility for RD research while providing a validated framework for TUNEL quantification in fluorescence microscopy. We have deposited it on GitHub.

## Methods

### Datasets

Three independent datasets were used to develop and validate the pipeline:

#### Dataset 1 (Primary/Training Dataset)

Fifty RD images from our laboratory, previously analyzed by both an experienced observer (>2-year experience) and an inexperienced observer (<1 year experience). Experimental RD was induced via ab interno subretinal injection of sodium hyaluronate in mouse eyes. Cryosections (10 µm) were stained using the ApoTag fluorescein direct in situ apoptosis detection kit (Millipore, Bedford, MA, USA) and counterstained with DAPI (Invitrogen #62248, Thermo Fisher Scientific, Waltham, MA, USA). Images were acquired using a fluorescence microscope (Axio Imager M2, Carl Zeiss) with a 20x/0.8 NA objective at 16-bit depth (1344 × 1024 resolution) targeting DAPI and Alexa Fluor 488 channels. Both observers performed manual counting in a masked fashion with duplicate images. All experiments complied with the ARVO Statement for the Use of Animals in Ophthalmic and Vision Research and were approved by the respective Institutional Animal Care and Use Committees.

#### Dataset 2 (Historical Dataset—Maidana et al.^10^)

Fifty images from a published RD study conducted approximately 10 years prior, served as temporal validation across different imaging conditions. Manual counting followed the masked protocol described in the original publication.^10^

#### Dataset 3 (External Validation—Murakami/Yamamoto)

Forty images provided by Drs. Yusuke Murakami and Kaho Yamamoto (Kyushu University, Japan), representing a geographically and methodologically independent validation cohort. Manual counting was performed by one observer from the collaborating laboratory in a masked fashion. RD was induced via ab externo subretinal injection of sodium hyaluronate as described by Matsumoto et al.^31^ This dataset required re-calibration of post-processing thresholds due to smaller TUNEL+ cell sizes. Parameters were calibrated using approximately five additional images separate from the 40-image validation cohort, selected for diversity in staining intensity and cell density, by inspecting the pipeline's visualization outputs. No further adjustments were made after this calibration step; the entire 40-image cohort therefore served as a fully independent held-out validation set (adjusted parameters: minimum area = 4 µm², maximum area = 15 µm², StarDist TUNEL threshold = 0.65, OF threshold = 0.65). The core deep learning models (U-Net and StarDist) were not retrained.

### Software Development and Architecture

RetinaDetachNet is implemented as a standalone Python application distributed via GitHub (https://github.com/kbaroutis/RetinaDetachNet). The software leverages Python-based deep learning libraries (StarDist 0.8.5, CSBDeep 0.7.4, scikit-image, NumPy, SciPy, pandas, and Matplotlib) within a conda environment for reproducible dependency management. Installation is automated through platform-specific scripts that create an isolated conda environment with all required dependencies. The application provides a cross-platform graphical user interface (GUI) with multi-threaded design for responsive processing.

The GUI features a parameter-driven workflow with eight configurable inputs (nuclei directory path, TUNEL directory path, pixels per micrometer, minimum area threshold, maximum area threshold, nuclei probability threshold, TUNEL probability threshold, and TUNEL overlap threshold) that manage every aspect of the analysis pipeline. A TUNEL overlap threshold parameter (default: 0.6) specifies the minimum fraction of each StarDist-segmented cell that must overlap with the binary threshold signal to be classified as TUNEL+, calculated as overlap fraction (OF = overlap area/StarDist cell area), reducing false positives caused by background noise or artifacts. The pipeline creates two output folders: (1) Visualizations/containing quality control images per sample (seven individual PNGs plus a combined 2 × 3 panel); and (2) Results/containing a comma-separated values (CSV) file with quantitative metrics (TUNEL count, nuclei count, ONL area, nuclei density).

### Image Analysis Pipeline

**Figure 1.**
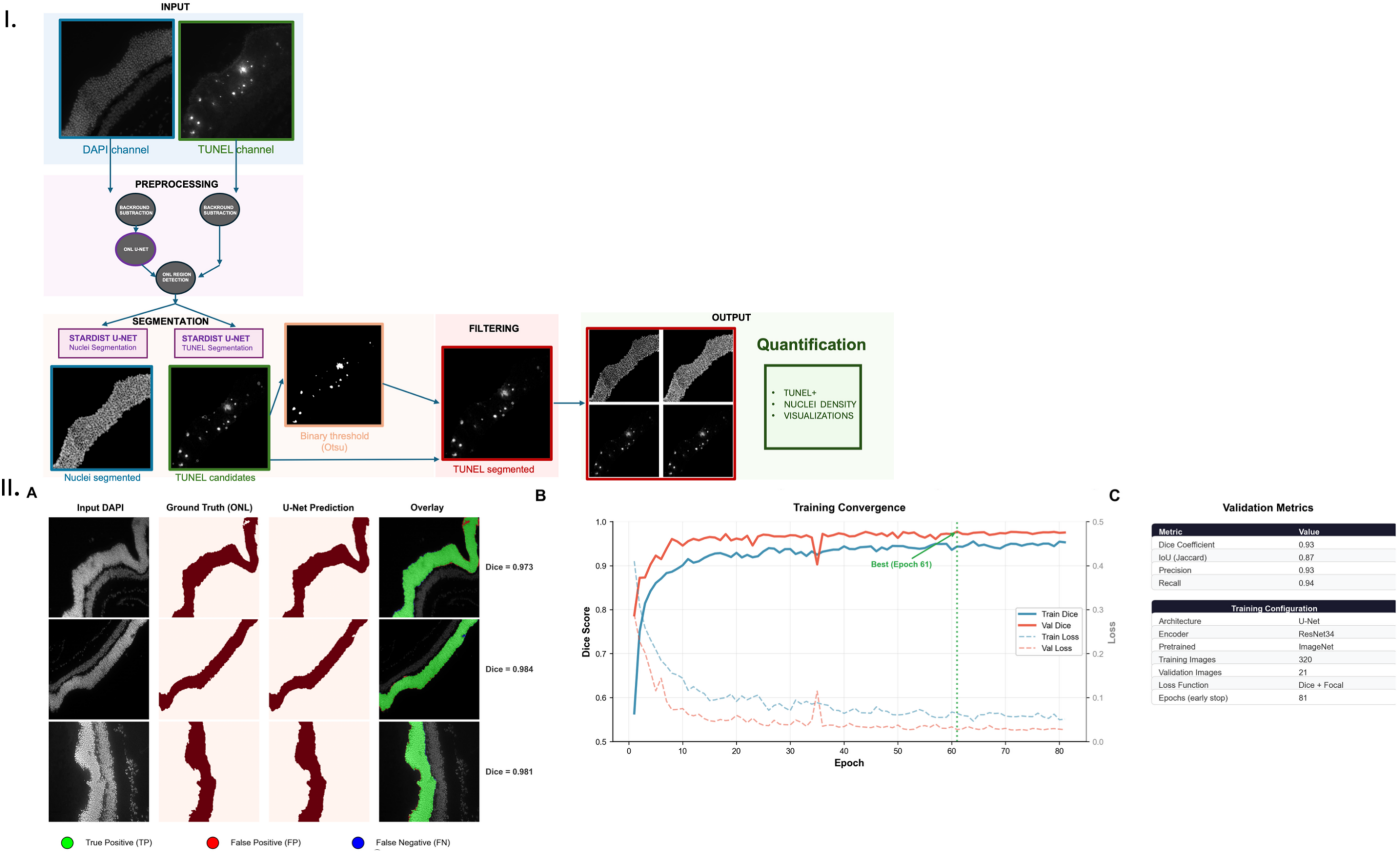
RetinaDetachNet analysis pipeline and U-Net ONL segmentation performance. (I) Pipeline architecture showing four sequential stages: ONL segmentation, background subtraction, deep learning-based cell segmentation, and TUNEL+ cell classification. (II) U-Net ONL segmentation validation: (**A**) Representative validation images showing input DAPI, ground truth ONL mask, predicted ONL mask, and overlay visualization (*green* = true positive; *red* = false positive; *blue* = false negative). (**B**) Training curves showing Dice score and loss convergence over 81 epochs with early stopping. (**C**) Validation metrics: Dice coefficient = 0.93, intersection-over-union (IoU) = 0.87, precision = 0.93, recall = 0.94 (*n* = 21 images).

The automated analysis pipeline consists of four sequential stages: (1) ONL segmentation, (2) background subtraction, (3) deep learning-based cell segmentation, and (4) TUNEL+ cell classification (Fig. 1).

#### Stage 1: ONL Segmentation

The ONL region of interest is automatically identified using a custom-trained U-Net model with a ResNet34 encoder pretrained on ImageNet.^21^ Ground truth ONL masks were manually delineated by a single trained observer, an ophthalmology researcher with specialized expertise in retinal layer morphology on 341 DAPI-stained images (320 training, 21 validation), consistent with established practice in retinal image segmentation.^32^^,^^33^ The model was trained on these annotations using binary segmentation (ONL vs. background). Training used a combined Dice and Focal loss function^34^ with positive class weighting (5.1) to address class imbalance (ONL represents 16.3% of pixels). Data augmentation included horizontal/vertical flips, rotations, elastic transforms, and brightness adjustments. The model outputs a probability map thresholded at 0.5 to generate binary ONL masks, enabling robust tissue boundary detection even in cases of severe retinal disruption where conventional thresholding approaches fail.

#### Stage 2: Background Subtraction

Rolling ball background subtraction^35^ (radius = 6.5 µm, automatically scaled based on pixel calibration) is applied to both DAPI and TUNEL channels to correct for uneven illumination and reduce autofluorescence artifacts.

#### Stage 3: Deep Learning Segmentation

Individual cells are segmented using the pretrained StarDist2D model (2D_versatile_fluo).^24^

#### Stage 4: TUNEL+ Cell Classification

A two-stage filtering approach combines morphological filtering (area-based) with overlap-based validation to identify true TUNEL+ cells. The StarDist model uses a U-Net architecture (depth = 3, 32 base filters, 32 radial directions) for predicting star-convex polygons from single-channel fluorescence images. Before StarDist processing, images were normalized using percentile-based normalization (1st-99.8th percentile) via the CSBDeep normalize() function.^23^ StarDist segmentation was performed with probability thresholds of 0.01 for nuclei and 0.5 for TUNEL channels (default parameters).

TUNEL+ cells were identified through a dual-filtering approach. First, segmented objects were filtered by area (10–60 µm² for the primary dataset) to exclude debris and cell clusters. Second, filtered cells were validated by calculating the OF with a binary threshold mask generated by applying Otsu's method^36^ to the background-subtracted and normalized TUNEL channel, where OF = (overlap area)/(StarDist cell area). Only cells with OF ≥ 0.6 were classified as TUNEL+.

### Statistical Analysis

Agreement between automated and manual quantification was assessed using the Spearman rank correlation coefficient (ρ), chosen for its robustness to outliers and non-linear relationships. Correlation analyses were performed separately for each dataset and for comparisons between different analysis methods (combined pipeline vs. Otsu-only vs. StarDist-only).

Method agreement was further evaluated using Bland-Altman analysis,^37^ which quantifies the mean difference (bias) and limits of agreement (mean difference ± 1.96 × standard deviation) between paired measurements (Supplementary Fig. S1). To assess the robustness and reliability of the correlation estimates, bootstrap resampling analysis was performed with 5000 iterations. For each iteration, samples were randomly drawn with replacement from the original dataset, and Spearman correlations were recalculated. The bootstrap distribution was used to compute 95% confidence intervals (CIs) using the percentile method (2.5th and 97.5th percentiles). Bootstrap analyses were conducted for all three analysis methods (combined pipeline, Otsu-only, StarDist-only) when compared against both experienced and inexperienced observers. All statistical analyses were performed using Python 3.8. Statistical significance was defined as *P* < 0.05 for all analyses.

## Results

### TUNEL Quantification Performance

#### Performance in the Primary Training Dataset

The primary dataset (Dataset 1) consisted of 50 RD images from our laboratory. Manual counting was performed independently by an experienced observer (>2 years of TUNEL quantification experience) and an inexperienced observer (<1 year of experience), both working in a masked fashion. The RetinaDetachNet demonstrated strong correlation with both observers: Spearman's ρ = 0.8703 (*P* < 0.0001) with the experienced observer and 0.8021 (*P* < 0.0001) with the inexperienced observer. Automated counts (mean ± standard deviation [SD]: 16.38 ± 11.54) were comparable to experienced observer counts (15.08 ± 8.55) and inexperienced observer counts (13.02 ± 6.25). Bland-Altman analysis confirmed minimal systematic bias between RetinaDetachNet and experienced observer counting (mean difference: 1.30 cells, limits of agreement: −11.71 to 14.31 cells), with no systematic trend across the measurement range.

**Figure 2.**
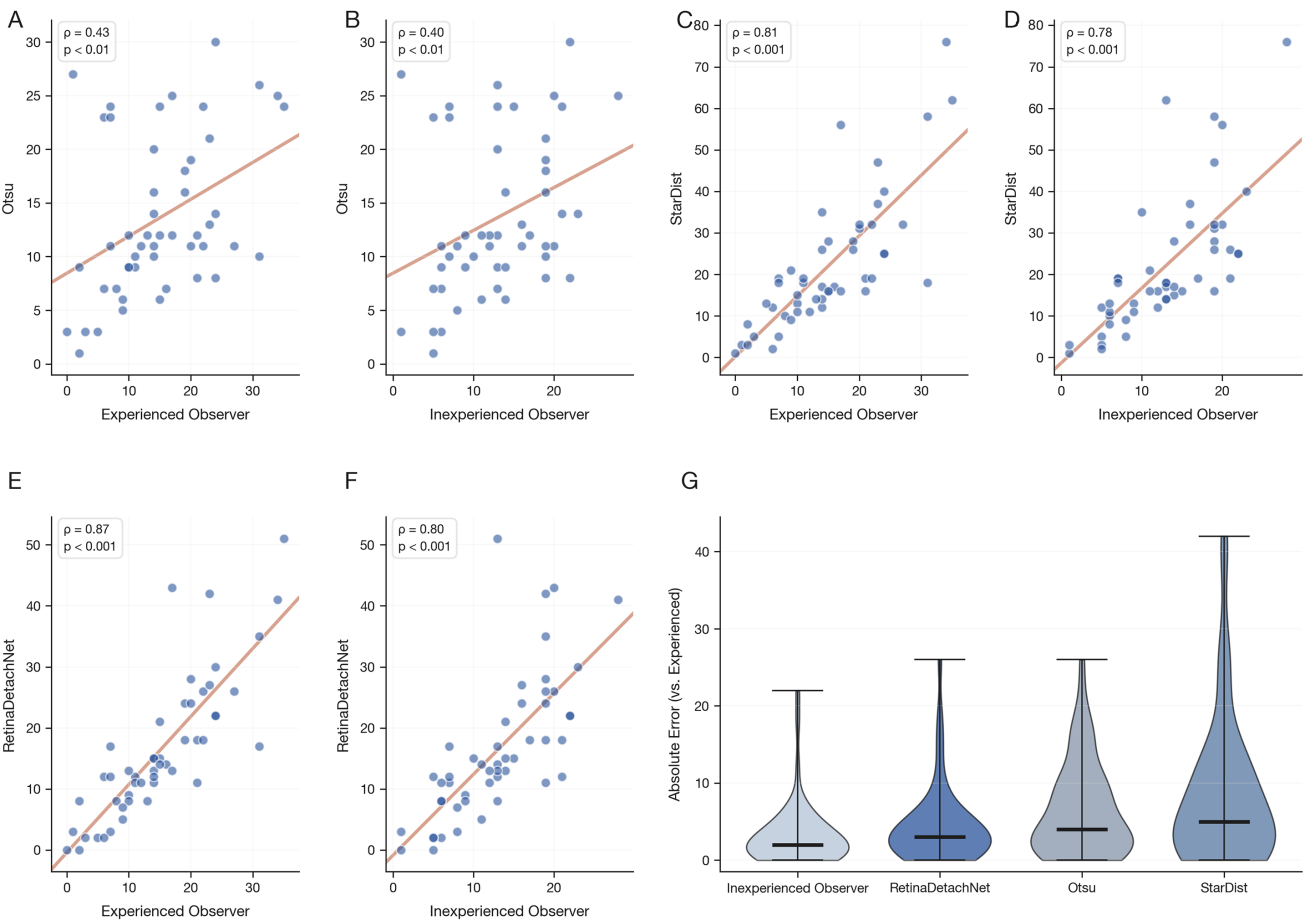
Correlation analysis and method comparison for Dataset 1 (primary training dataset). (**A**–**F**) Spearman correlation scatter plots comparing RetinaDetachNet, StarDist-only, and Otsu-only methods against experienced and inexperienced observers. (**G**) Violin plots of absolute error distributions across methods (median [IQR]: RetinaDetachNet, 3 [1–5]; Otsu, 4 [2–9]; StarDist, 5 [1.3–12] cells).

To assess the relative performance of different analysis approaches, we compared the combined RetinaDetachNet pipeline (U-Net for ONL segmentation followed by StarDist cell detection with overlap filtering) against Otsu-only and StarDist-only methods for TUNEL detection. The combined pipeline substantially outperformed the Otsu-only approach (ρ = 0.4284, *P* = 1.91 × 10^−^³) and performed comparably to the StarDist-only method (ρ = 0.8052, *P* = 1.80 × 10^−^¹²) (Fig. 2).

#### Validation on a Historical Dataset From a Previously Published Study

Dataset 2 comprised 50 images from a published RD study conducted approximately 10 years prior.^10^

The RetinaDetachNet achieved exceptional correlation with the experienced observer (Spearman ρ = 0.9826, *P* < 0.0001), demonstrating robustness across temporal and methodological variations. Correlation with the inexperienced observer was also strong (ρ = 0.9090, *P* < 0.0001). Automated counts (26.32 ± 20.44) closely matched experienced observer counts (27.28 ± 17.20), whereas the divergence from inexperienced observer counts (32.62 ± 18.91) was greater. Bland-Altman analysis demonstrated exceptional agreement between RetinaDetachNet and experienced observer, with near-zero bias (mean difference: −0.96 cells) and tight limits of agreement (−9.86 to 7.94 cells).

**Figure 3.**
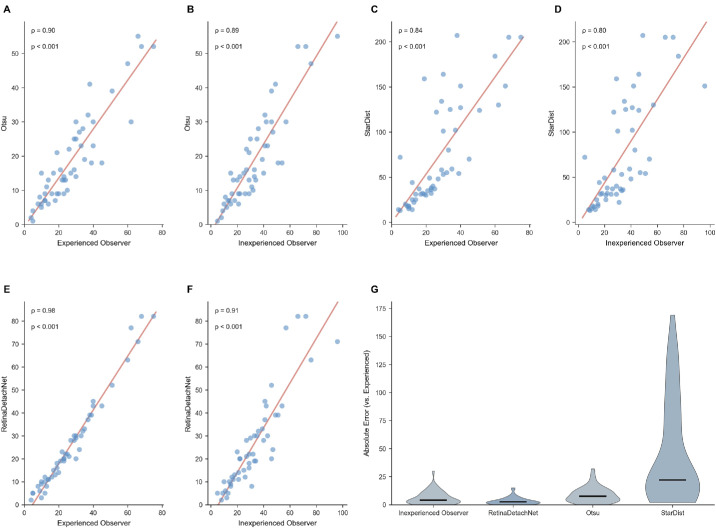
Validation on historical dataset (Dataset 2—Maidana et al.^10^). (**A**–**F**) Spearman correlation scatter plots for temporal validation on 50 images from a study conducted ∼10 years prior. (**G**) Violin plots of absolute error distributions across methods (median [IQR]: RetinaDetachNet, 2.5 [1–4.8]; Otsu, 7.5 [4–12]; StarDist, 22 [12–72.5] cells).

Method comparison revealed the superiority of the combined approach over individual segmentation methods. Otsu thresholding^36^ alone achieved moderate correlation (ρ = 0.8993, *P* < 0.0001) but systematically undercounted cells (18.78 ± 13.50), with a mean difference of −8.50 cells compared to manual counting. StarDist-only segmentation without overlaps filtering showed a strong correlation (ρ = 0.8419, *P* < 0.0001), but substantially overcounted cells (72.88 ± 58.40), with a mean difference of 45.60 cells. The combined RetinaDetachNet pipeline, incorporating both deep learning segmentation and overlap-based filtering, achieved optimal performance (ρ = 0.9826) with minimal systematic bias (−0.96 cells) (Fig. 3).

#### Validation on an External Dataset With Parameter Calibration

Dataset 3 consisted of 40 images from Drs. Murakami and Yamamoto (see Methods), representing a geographically and methodologically independent external validation cohort. We adjusted the minimum area threshold from 10 µm² to 4 µm², the maximum area threshold from 60 µm² to 15 µm², the StarDist TUNEL probability threshold from 0.5 to 0.65, and the OF threshold from 0.6 to 0.65. These adjustments accounted for smaller TUNEL+ cell sizes characteristic of this dataset.

**Figure 4.**
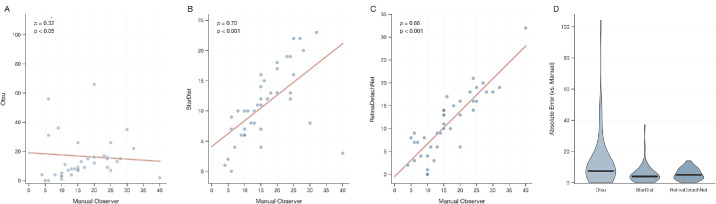
External validation with parameter calibration (Dataset 3—Murakami/Yamamoto). (**A**–**C**) Spearman correlations for Otsu-only (ρ = 0.32), StarDist-only (ρ = 0.70), and RetinaDetachNet (ρ = 0.86) on 40 images with calibrated parameters. (**D**) Violin plots of absolute errors (median [IQR]: RetinaDetachNet, 5 [3–8.3]; Otsu, 7.5 [5–11.5]; StarDist, 4 [3–6.3] cells).

Consistent with the method comparisons performed across all validation datasets, Dataset 3 demonstrated that RetinaDetachNet achieved the strongest correlation with manual counting (Spearman ρ = 0.8564, *P* = 1.84 × 10^−^¹²), substantially outperforming both StarDist-only (ρ = 0.7048, *P* = 3.83 × 10^−^⁷) and Otsu-only (ρ = 0.3249, *P* = 0.0408) approaches on 40 matched samples. RetinaDetachNet demonstrated superior consistency with automated counts of 11.22 ± 6.89 cells versus manual counts of 16.43 ± 8.34 cells (mean difference: −5.21 ± 4.16 cells). In contrast, the Otsu-only method exhibited high variability (16.62 ± 20.58 cells) and poor agreement with manual counts (mean absolute difference: 13.25 cells). In comparison, the StarDist-only method showed intermediate performance (11.08 ± 6.05 cells, mean difference: −5.68 cells). Although StarDist-only exhibited a marginally lower median absolute error than RetinaDetachNet in this dataset (median: four vs. five cells), its substantially lower correlation (ρ = 0.70 vs. 0.86) indicates poorer rank consistency across the measurement range, which is the primary metric for evaluating between-group treatment effects in neuroprotection studies. Bland-Altman analysis confirmed that this underestimation represents a fixed offset rather than a proportional bias, because the difference did not scale with cell count magnitude (mean difference: −5.21 cells, limits of agreement: −13.46 to 3.06 cells; Supplementary Fig. S1C) (Fig. 4).

### Bootstrap Analysis Confirms the Robustness of the Quantification Methods

Across all three datasets, bootstrap analysis confirmed the superior performance and reliability of the RetinaDetachNet combined pipeline (Table). In the primary training dataset (*n* = 50), the combined pipeline showed strong correlation with experienced manual counting (bootstrap mean Spearman ρ = 0.8624; 95% CI, 0.7651–0.9307; *P* = 2.24 × 10^−^¹⁶) and comparable performance against inexperienced counting (bootstrap mean ρ = 0.8527; 95% CI, 0.7272–0.9346; *P* = 9.89 × 10^−^¹⁶). The relatively narrow CIs indicate high stability of these correlation estimates.

**Table.** Comparison of Dataset Characteristics and RetinaDetachNet Performance Across Three Independent Validation Cohorts

Characteristic	Dataset 1	Dataset 2	Dataset 3
Dataset Information			
Sample size (n)	50	50	40
Source laboratory	Massachusetts, USA	Massachusetts, USA	Japan
Temporal characteristics	Recent (training set)	Historical (∼10 years prior)	Recent (external validation)
Manual observers	Experienced + Inexperienced	Experienced + Inexperienced	Manual counting
Manual Counting Results (mean ± SD)			
Experienced observer	15.08 ± 8.55 cells	27.28 ± 17.20 cells	16.43 ± 8.34 cells
Inexperienced observer	13.02 ± 6.25 cells	32.62 ± 18.91 cells	N/A
RetinaDetachNet Performance			
Automated count (mean ± SD)	16.38 ± 11.54 cells	26.32 ± 20.44 cells	11.22 ± 6.89 cells
Spearman ρ (observed)	0.8703	0.9826	0.8564
P-value	2.24 × 10^−16^	9.73 × 10^−37^	1.84 × 10^−12^
Bootstrap Spearman ρ (95% CI)	0.862 [0.765, 0.931]	0.979 [0.956, 0.993]	0.845 [0.715, 0.928]
Method Comparison vs. Manual			
Otsu-only Spearman ρ	0.4284	0.8993	0.3249
StarDist-only Spearman ρ	0.8052	0.8419	0.7048
Optimized Parameters			
Min area (µm^2^)	10	10	4
Max area (µm^2^)	60	60	15
StarDist TUNEL threshold	0.5	0.5	0.65
TUNEL overlap fraction (OF) threshold	0.6	0.6	0.65
Key Findings			
Validation type	Training & internal validation	Temporal validation	Geographic & methodological validation
Performance vs. individual methods	Superior to Otsu-only; comparable to StarDist-only	Superior to both Otsu-only and StarDist-only	Superior to both Otsu-only and StarDist-only

CI, confidence interval; N/A, not available; OF, overlap fraction; SD, standard deviation.

Note: Dataset 1 served as the primary training set for parameter optimization. Dataset 2 (Maidana et al.) represents a historical validation cohort from the same laboratory approximately 10 years prior, testing robustness across temporal and methodological variations. Dataset 3 (Japan) represents an external validation cohort requiring parameter re-optimization for smaller TUNEL-positive cell morphology. All correlations compare automated RetinaDetachNet counts to experienced observer manual counts unless otherwise specified.

The historical validation dataset (*n* = 50) demonstrated exceptional bootstrap-validated performance (ρ = 0.9789; 95% CI, 0.9556–0.9925), exceeding interobserver agreement (ρ = 0.9352). The external validation cohort (Dataset 3, *n* = 40) showed robust correlation (ρ = 0.8450; 95% CI, 0.7148–0.9275) despite methodological differences between laboratories.

Comparison of different analysis methods across all datasets revealed substantial and consistent performance differences. The Otsu-only method showed highly dataset-dependent performance, ranging from weak correlation in the external validation cohort (bootstrap mean ρ = 0.3265; 95% CI, −0.0734 to 0.6933; *P* = 4.08 × 10^−^²) to moderate correlation in the primary dataset (bootstrap mean ρ = 0.4267; 95% CI, 0.1264–0.6830; *P* = 1.91 × 10^−^³) and strong correlation in the historical dataset (bootstrap mean ρ = 0.8903; 95% CI, 0.8078–0.9461; *P* = 7.21 × 10^−^¹⁹). The wide variability and broader confidence intervals indicate that conventional thresholding alone is not robust across different imaging conditions.

**Figure 5.**
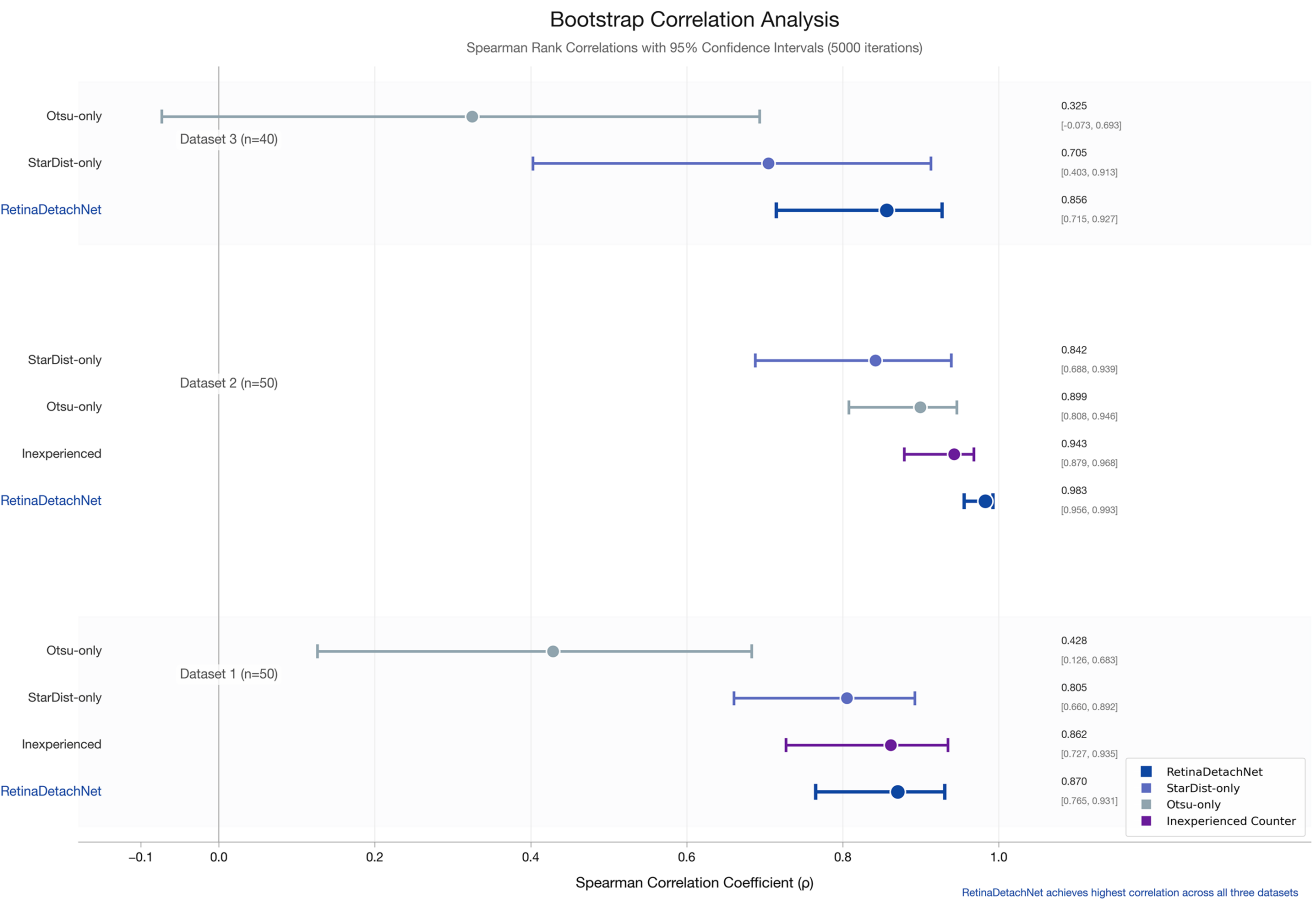
Bootstrap correlation analysis across all datasets and methods. Forest plot of bootstrap-validated Spearman correlations with 95% confidence intervals (5,000 iterations). RetinaDetachNet demonstrates consistent superior performance (ρ = 0.85–0.98) compared to Otsu-only (ρ = 0.33–0.90) and StarDist-only (ρ = 0.70–0.84) across all datasets.

The StarDist-only method consistently achieved moderate-to-strong correlations across all datasets: primary dataset (bootstrap mean ρ = 0.7962; 95% CI, 0.6605–0.8923; *P* = 1.80 × 10^−^¹²), historical dataset (bootstrap mean ρ = 0.8326; 95% CI, 0.6878–0.9392; *P* = 1.86 × 10^−^¹⁴), and external validation cohort (bootstrap mean ρ = 0.6968; 95% CI, 0.4026–0.9130; *P* = 3.83 × 10^−^⁷). Although StarDist-only showed more consistent performance than Otsu-only, it was consistently outperformed by the combined RetinaDetachNet pipeline across all datasets (Fig. 5).

### U-Net ONL Segmentation Performance

The custom-trained U-Net model for ONL segmentation was validated on a held-out set of 21 images. The model achieved a Dice coefficient of 0.93 and intersection-over-union of 0.87, with precision of 0.93 and recall of 0.94. Training converged after 81 epochs with early stopping, demonstrating stable learning without overfitting. These metrics indicate robust binary segmentation performance, with balanced precision-recall suggesting minimal over-segmentation or under-segmentation of the ONL region (Fig. 1II).

### Nuclei Density Quantification

In addition to TUNEL+ cell quantification, the RetinaDetachNet automatically quantifies total nuclei density within the segmented ONL region. To validate nuclear counting accuracy, we compared automated nuclear counts with manual quantification by an experienced observer across eight images from Dataset 1. The automated nuclei counting demonstrated perfect rank correlation with manual counting (Spearman ρ = 1, *P* < 0.0001), indicating identical ranking of all images from lowest to highest nuclei density. The mean difference between automated and manual counts was 6.38 nuclei (SD = 16.16), with a mean relative error of 6.10%, demonstrating excellent absolute agreement. Automated counts (mean ± SD: 160.75 ± 95.46) were comparable to manual counts (154.38 ± 91.98) (Supplementary Fig. S2).

## Discussion

To our knowledge, RetinaDetachNet is the first deep learning-based pipeline specifically designed and validated for automated TUNEL quantification in experimental RD, with three principal contributions: (1) a dual deep learning architecture integrating custom-trained U-Net for tissue segmentation with StarDist for cell detection; (2) a novel U-Net model achieving robust ONL boundary detection (Dice coefficient = 0.93) that enables accurate region-of-interest definition even in severely disrupted retinal tissue; and (3) strong correlation with manual counting validated across three independent datasets spanning temporal, geographic, and methodological variations.

Notably, in the historical validation dataset, RetinaDetachNet achieved a correlation of ρ = 0.98 with the experienced observer, surpassing inter-observer agreement between experienced human raters (ρ = 0.94). This result suggests that the automated pipeline performs at or above the ceiling of human reproducibility, achieving expert-level equivalence in TUNEL quantification.

The need for automated quantification in experimental RD is pressing. TUNEL counting remains the principal endpoint for evaluating photoreceptor cell death in neuroprotection studies, yet manual quantification requires trained observers working in masked fashion with duplicate counts, a labor-intensive process prone to inter- and intra-observer variability that limits reproducibility across laboratories.^13^^–^^15^ Parallel efforts in adjacent fields, such as RGCode for retinal ganglion cell quantification in glaucoma,^30^ underscore the broader demand for standardized, observer-independent tools in preclinical ophthalmic research.

Our findings align with the broader literature demonstrating that deep learning techniques overcome the limitations of traditional image processing methods.^18^^,^^19^^,^^38^ Conventional thresholding approaches struggle with noisy or variable images, leading to low accuracy and high noise sensitivity.^17^ In our study, the Otsu-only method exhibited highly dataset-dependent performance (ρ = 0.33 to 0.90) with wide CIs, reflecting the fundamental limitation of fixed-threshold approaches when confronting variations in staining intensity, background fluorescence, and tissue morphology across laboratories and time periods.

The star-convex polygon representation employed by StarDist is inherently well-suited for approximating the roundish morphology of cell nuclei,^24^ but requires additional validation steps to distinguish true TUNEL+ cells from artifacts and debris. Notably, StarDist-only analysis exhibited dataset-dependent bias direction: substantial overcounting in Dataset 2 (mean difference: 45.60 cells) versus undercounting in Dataset 3 (mean difference: −5.68 cells). This reversal likely reflects differences in TUNEL+ cell morphology and staining intensity between cohorts. Dataset 2's higher background fluorescence and debris content led to false positive detections, whereas Dataset 3's smaller TUNEL-positive cell bodies combined with the calibrated higher probability threshold (0.65 vs. 0.50 default) reduced false positives but may have excluded genuine dim-positive cells. These findings underscore the necessity of the dual-filtering approach of RetinaDetachNet to achieve consistent performance regardless of dataset-specific imaging characteristics.

Although alternative deep learning approaches exist for cell segmentation, including Cellpose^27^^,^^28^ and nnU-Net,^22^ we selected StarDist for TUNEL+ cell detection based on its specific optimization for nuclear segmentation in fluorescence microscopy and its demonstrated performance across diverse applications.^24^^–^^26^^,^^29^ We used StarDist and not Cellpose, which uses gradient flow tracking for whole-cell segmentation, due to the speed that StarDist offers for the same accuracy. The modular architecture of RetinaDetachNet does permit integration of alternative segmentation if one prefers alternative segmentation modules.

The RetinaDetachNet pipeline extends earlier work^10^ by incorporating deep learning-based cell segmentation, which offers several advantages: (1) superior handling of touching or overlapping cells through star-convex polygon representation, (2) robust performance across variable imaging conditions without requiring parameter adjustment for each image, (3) pretrained models leveraging large datasets that enhance generalization, and (4) state-of-the-art segmentation accuracy validated across diverse biomedical applications.^24^^–^^26^ Our methodology parallels recent advances in automated retinal cell quantification, including RGCode, a U-Net-based pipeline for retinal ganglion cell quantification in murine retina,^30^ demonstrating the broader applicability of deep learning approaches for automated cell counting in ophthalmic research.

Several technical considerations warrant discussion. First, while the custom-trained U-Net demonstrates robust ONL segmentation performance, cases of severe retinal disruption may still require manual quality control (Supplementary Fig. S3); additionally, ONL ground truth masks were generated by a single annotator, and inter-annotator variability was not formally assessed. Second, adapting the pipeline to new datasets requires calibration of post-processing thresholds on approximately five images separate from the validation cohort; whereas the core deep learning models remain frozen, the risk of dataset-specific threshold overfitting should be acknowledged. Third, Bland-Altman analyses confirmed near-zero bias for Datasets 1 and 2, but Dataset 3 showed a fixed-offset underestimation (mean difference: −5.21 cells, LoA: −13.46 to 3.06); within-dataset comparisons are unaffected, although absolute cross-study pooling requires dataset-specific calibration. Fourth, validation relies on agreement with manual counting a relative rather than absolute reference standard, though the pipeline surpassing interobserver agreement (ρ = 0.98 vs. ρ = 0.94) suggests performance at the ceiling of the available standard. While validation across 140 images from three independent cohorts provides robust evidence, expanding to additional institutions would further strengthen generalizability.

Various avenues for future development emerge from this work. First, extending the pipeline to 3D volumetric analysis could enable comprehensive quantification across entire tissue sections or whole-mount retinas, leveraging recent advances in 3D cell segmentation.^39^^–^^41^ Second, developing predictive models that distinguish cell death modalities based on morphological features could extend beyond TUNEL-based detection to capture multiple forms of regulated cell death simultaneously.^11^ Additionally, integration with deep learning-based image quality assessment could enable automated detection of technical artifacts, staining inconsistencies, or tissue damage that might compromise quantification accuracy. Finally, multi-annotator consensus protocols (e.g., STAPLE-based fusion) and synthetic ground truth datasets with programmatically defined cell counts^29^^,^^42^ could provide more rigorous validation benchmarks.

## Conclusions

To our knowledge, RetinaDetachNet represents the first validated deep learning-based pipeline for automated TUNEL quantification in experimental RD, addressing a significant methodological gap in retinal research. It outperforms individual methods across temporal, geographic, and methodological variations. Strong correlation with manual counting, high reproducibility demonstrated through bootstrap analysis, and superior performance compared to traditional thresholding or deep learning alone suggest that RetinaDetachNet can serve as a robust and generalizable tool for retinal detachment researchers.

## Supplementary Material

Supplement 1
