# Supplementary material for: RetinaDetachNet: Automated Deep Learning Quantification of Photoreceptor Cell Death for Neuroprotection Studies in Experimental Retinal Detachment
Source: Transl Vis Sci Technol. 2026 Apr 22;15(4):23. doi: 10.1167/tvst.15.4.23 (PMC13107991; doi:10.1167/tvst.15.4.23)
Supplement: Supplement 1 [file tvst-15-4-23_s001.pdf]

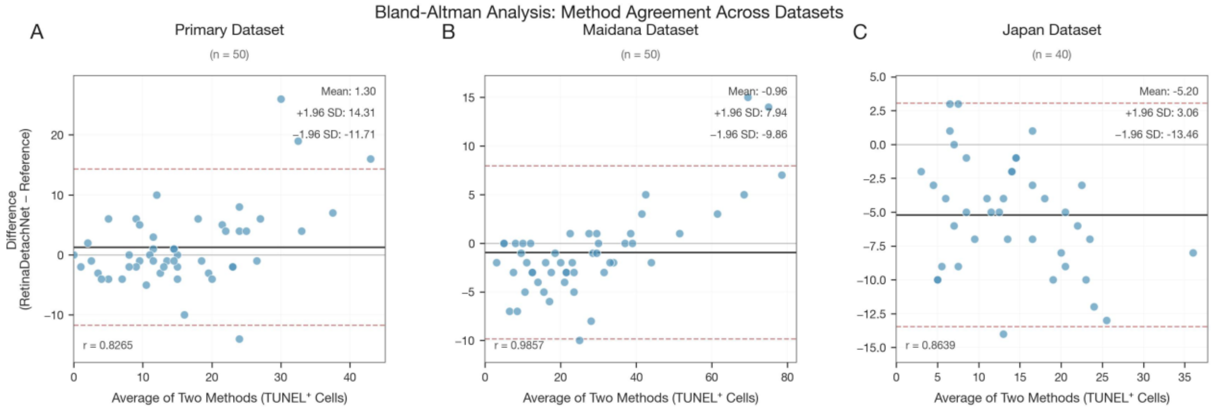

Supplementary Figure 1. Bland-Altman Analysis of Method Agreement Across Datasets. (A) Primary dataset (mean difference = 1.30 cells). (B) Maidana dataset (mean difference = -0.96 cells). (C) Dataset 3 (mean difference = -5.20 cells). Dashed lines indicate bias; dotted lines indicate ±1.96 SD limits of agreement.

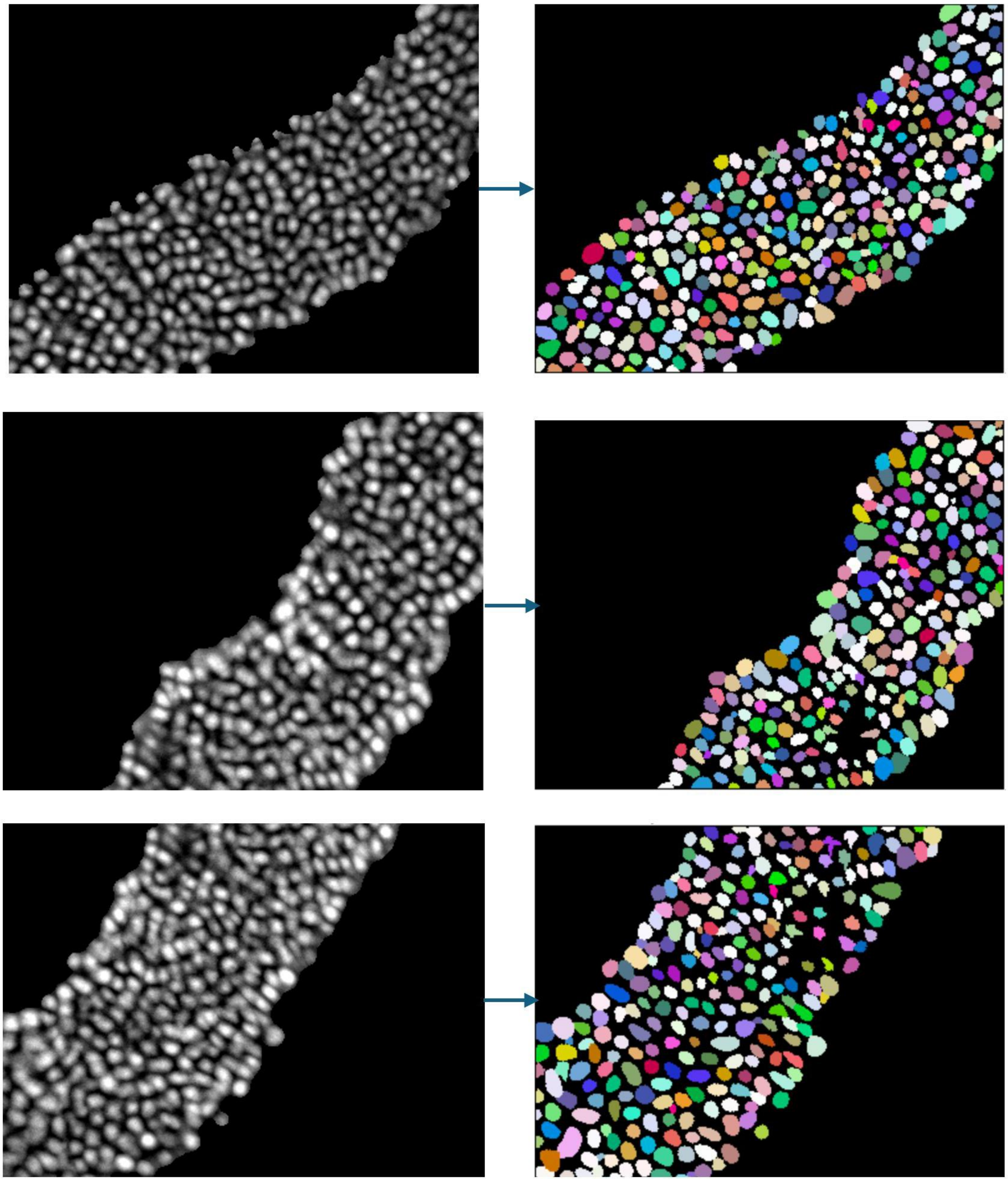

Supplementary Figure 2. Nuclei Density Quantification Validation. Example nuclei segmentation and correlation analysis comparing automated and manual counting across 8 images. Perfect rank correlation (Spearman $\rho = 1$, $p < 0.0001$) with a mean relative error of 6.10%.

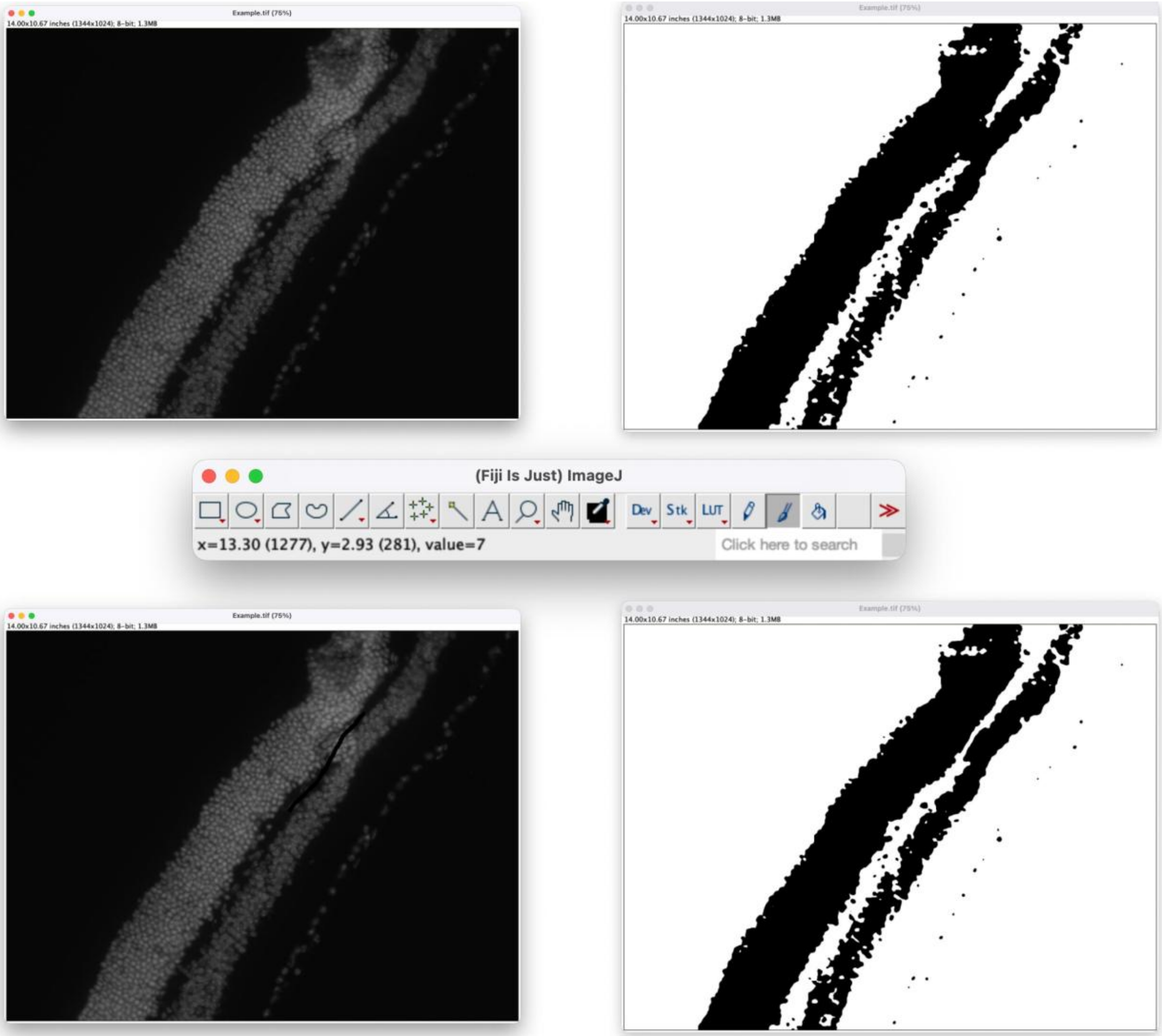

Supplementary Figure 3. Pipeline Limitation in Severely Disrupted Retinal Tissue. Example of ONL segmentation failure in severe retinal detachment with highly disrupted architecture, requiring manual quality control (with proposed solution).
